# Sex disparities in Hispanic melanoma patients: A retrospective cohort analysis of the 2000–2020 Surveillance, Epidemiology and End Results database

**DOI:** 10.1002/ski2.415

**Published:** 2024-06-25

**Authors:** Mitchell A. Taylor, Anjali Mishra, Bianca Ituarte, Michaela Clausen, Erin X. Wei

**Affiliations:** ^1^ Department of Dermatology University of Nebraska Medical Center Omaha Nebraska USA; ^2^ Creighton University School of Medicine Omaha Nebraska USA; ^3^ University of Missouri‐Kansas City School of Medicine Kansas City Missouri USA

## ETHICS STATEMENT

Review by the University of Nebraska Medical Center Institutional Review Board (IRB) was not deemed necessary for this study.

## PATIENT CONSENT

Not applicable.

Dear Editor,

Melanoma ranks among the most common malignancies in the United States, with an estimated 100 640 new melanoma cases projected for 2024.^1^ Previous studies analysing melanoma‐specific survival have established poor outcomes in Hispanic patients compared to non‐Hispanic White individuals.^2^ However, there is a paucity of literature investigating sex differences in disease presentation and outcomes in Hispanic individuals diagnosed with cutaneous melanoma. Thus, the purpose of this study is to further examine differences in melanoma characteristics and disease‐specific survival (DSS) between Hispanic males and females diagnosed with cutaneous melanoma.

In this retrospective cohort study, the Surveillance, Epidemiology, and End Results database was queried to identify Hispanic individuals of any race diagnosed with biopsy‐proven cases of cutaneous melanoma (ICD‐O‐3 histology codes 8720/3–8780/3; primary site codes C44.0–C44.9) from 2000 to 2020. Statistical tests including Chi‐squared, Mann–Whitney *U*, Kaplan–Meier and log‐rank, and multivariate Cox proportional hazards were conducted using SPSS version 29 (significance *p* < 0.05).

Between 2000 and 2020, 12 328 cases were identified, with 43.3% identifying as male and 56.7% identifying as female (Table 1). There was a significant association between sex and age (*p* < 0.001), where the greatest number of males were aged 60–79 (39.7%) and females were aged 40–59 (39.0%). There was also a significant association between sex and primary tumour site (*p* < 0.001), with the most common primary site being the trunk for males (31.4%) and the lower extremity for females (35.2%). Females were diagnosed with localized disease at higher rates (80.3% vs. 70.2%), while males were diagnosed with regional (18.5% vs. 13.8%) and distant disease (11.3% vs. 5.9%) (*p* < 0.001). Men exhibited higher proportions of tumours with increased Breslow thickness in the ranges of 1.01–2.00 mm (16.4% vs. 15.3%), 2.01–4.00 mm (14.9% vs. 10.9%), and >4.00 mm (16.6% vs. 11.4%) compared to women (*p* < 0.001), as well as higher rates of regional lymph node positivity (16.2% vs. 10.6%; *p* < 0.001).

**TABLE 1:** Clinical‐pathological features of Hispanic melanoma cohort by sex.

Variable	Male (*n* = 5334)	Female (*n* = 6994)	*p*‐value
Age at diagnosis (years)			
0–19	73 (1.4%)	117 (1.7%)	**<0.001**
20–39	646 (12.1%)	1462 (20.9%)	
40–59	1824 (34.2%)	2730 (39.0%)	
60–79	2116 (39.7%)	2105 (30.1%)	
80+	675 (12.7%)	580 (8.3%)	
Primary tumour site			
Head & neck	1285 (26.1%)	1068 (16.0%)	**<0.001**
Trunk	1545 (31.4%)	1547 (23.2%)	
Upper extremity	952 (19.3%)	1713 (25.7%)	
Lower extremity	1145 (23.2%)	2349 (35.2%)	
Melanoma subtype			
Lentigo maligna	237 (4.4%)	202 (2.9%)	**<0.001**
Superficial spreading	1263 (23.7%)	2057 (29.4%)	
Acral lentiginous	291 (5.5%)	322 (4.6%)	
Nodular	577 (10.8%)	554 (7.9%)	
Other/NOS	2966 (55.6%)	3859 (55.2%)	
Disease stage^a^			
Localized	3100 (70.2%)	4536 (80.3%)	**<0.001**
Regional	819 (18.5%)	777 (13.8%)	
Distant	500 (11.3%)	333 (5.9%)	
Regional lymph node positivity^b^			
Yes	822 (16.2%)	706 (10.6%)	**<0.001**
No	1380 (27.1%)	1888 (28.3%)	
Not examined	2883 (56.7%)	6662 (61.1%)	
Breslow thickness (mm)			
≤1.00	1444 (52.1%)	2321 (62.4%)	**<0.001**
1.01–2.00	455 (16.4%)	568 (15.3%)	
2.01–4.00	414 (14.9%)	407 (10.9%)	
>4.00	459 (16.6%)	425 (11.4%)	
Surgical treatment^c^	4522 (85.8%)	6155 (89.0%)	**<0.001**
Sentinel lymph node surgery^c^	1954 (41.2%)	2298 (37.0%)	**<0.001**
Chemotherapy^c^	257 (4.8%)	200 (2.9%)	**<0.001**
Radiation^c^	286 (5.4%)	179 (2.6%)	**<0.001**

*Note*: Significant *p*‐values (<0.05) are in bold.

Abbreviation: NOS, not otherwise specified; SEER, Surveillance, Epidemiology and End Results.

^a^ The SEER historical staging system categorizes malignancies as either localized (restricted to the organ of origin), regional (spreading beyond the originating organ or involving nearby lymph nodes), or distant (having spread to a distant part of the body through direct extension, non‐contiguous metastasis, or via the lymphatic system to distant lymph nodes). This classification framework is applicable across a broad spectrum of malignancies and accommodates variations in formal staging systems over time.

^b^ Number of regional nodes examined by the pathologist and found to contain metastases.

^c^ In comparison to not receiving this treatment or surgery.

On conducting univariate Kaplan–Meier analysis, males demonstrated significantly reduced 5‐ and 10‐year DSS rates (75.0% and 66.0%, respectively) compared to females (86.0% and 81.0%, respectively; *p* < 0.001). After adjusting for potential confounders including age, race, disease stage, annual income, rural versus urban living, and primary tumour site, male sex independently increased disease‐specific mortality compared to females (reference: females; adjusted hazard ratio 1.36; 95% CI 1.23–1.51; *p* < 0.001).

In this study, we provide an in‐depth analysis of sex disparities in Hispanic patients diagnosed with cutaneous melanoma. Consistent with previous literature examining overall melanoma sex differences,^3^, ^4^ our study found that Hispanic males also tend to receive melanoma diagnoses at an older age, exhibit advanced‐stage disease, and present with melanoma of the trunk compared to lower extremities for females. Furthermore, our findings indicate a significantly reduced DSS in Hispanic males compared to females, even after adjusting for potential confounders. This observation aligns with previous research studies on sex differences in melanoma, although such disparities in DSS have not been specifically discussed for Hispanic individuals. There appears to be an interplay of behavioural factors and immunological, hormonal, or genetic causes that may be contributing to the observed sex differences.^5^ These results underscore the importance of tailored interventions, targeted screening strategies, and enhanced educational initiatives to address the unique needs of Hispanic individuals, particularly males, in mitigating the burden of melanoma within this population. Future research and interventions are warranted to improve early detection, access to care, and ultimately survival outcomes among Hispanic individuals diagnosed with cutaneous melanoma.

Limitations to this study include missing data regarding immunotherapy and use of a national database, which may not fully represent the broader Hispanic population in the United States. Nevertheless, this study highlights important tumour behaviours in the Hispanic population, emphasizing sex‐specific differences in presentation and DSS.

## CONFLICT OF INTEREST STATEMENT

None to declare.

## AUTHOR CONTRIBUTIONS

**Mitchell A. Taylor**: Conceptualization (equal); data curation (equal); formal analysis (lead); investigation (equal); methodology (equal); software (equal); visualization (equal); writing – original draft (equal); writing – review & editing (equal). **Anjali Mishra**: Conceptualization (equal); data curation (equal); formal analysis (supporting); investigation (equal); methodology (equal); validation (equal); visualization (equal); writing – original draft (equal); writing – review & editing (equal). **Bianca Ituarte**: Conceptualization (supporting); methodology (supporting); validation (supporting); writing – review & editing (supporting). **Michaela Clausen**: Data curation (supporting); methodology (supporting); writing – original draft (supporting); writing – review & editing (supporting). **Erin X. Wei**: Conceptualization (supporting); formal analysis (supporting); investigation (supporting); methodology (supporting); resources (supporting); supervision (lead); validation (lead); visualization (supporting); writing – original draft (supporting); writing – review & editing (supporting).

## FUNDING INFORMATION

This article received no specific grant from any funding agency in the public, commercial, or not‐for‐profit sectors.

## Data Availability

The data underlying this article will be shared on reasonable request to the corresponding author.
